# Perceived Social Support and Help-seeking among U.S. Chinese Older Adults with Elder Mistreatment

**DOI:** 10.1093/geroni/igaa057.3215

**Published:** 2020-12-16

**Authors:** Ying-Yu Chao, Dexia Kong, XinQi Dong

**Affiliations:** 1 Rutgers School of Nursing, Newark, New Jersey, United States; 2 Rutgers University, New Brunswick, New Jersey, United States

## Abstract

Background/Purpose: Older immigrants are at risk of experiencing marginalization and social exclusion. Traditional Chinese culture values could deeply influence the older Chinese immigrants’ perceptions regarding mistreatment and motivating them to seek help. This study aimed to examine the associations between perceived social support and informal/formal help-seeking intentions and behaviors among U.S. Chinese older adults experiencing elder mistreatment. Methods: Data derived from the Population Study of Chinese Elderly in Chicago (PINE). Independent variables were positive and negative perceived social support. Dependent variables were informal/formal help-seeking intentions and behaviors. Descriptive statistics and logistic regression analyses were performed. Results: A total of 423 participants experienced elder mistreatment (mean age: 72.4 ±7.88 years old). The most common informal help-seeking sources were adult children, followed by partner, and friends/neighbors/colleagues. The most common sources of formal help-seeking were community social services organizations and the legal criminal justice system. After controlling for covariates, positive perceived social support was associated with informal help-seeking intentions (OR=1.14, 95% CI: 1.05-1.24, p < .01) and behaviors (OR=1.12, 95% CI: 1.04-1.22, p < .01). However, the associations between perceived social support and formal help-seeking intentions and behaviors were not significant. Conclusions & Implications: Further research is needed to examine the mediating effects of cultural values on the relationship between perceived social support and help-seeking among mistreated older Chinese immigrants. In addition, additional studies are needed to identify impede or facilitate factors of informal/formal elder mistreatment help-seeking. Prevention and intervention programs should incorporate valuable cultural insight to improve help-seeking among this population.

